# A high-resolution linkage map for comparative genome analysis and QTL fine mapping in Asian seabass, *Lates calcarifer*

**DOI:** 10.1186/1471-2164-12-174

**Published:** 2011-04-02

**Authors:** Chun Ming Wang, Zhi Yi Bai, Xiao Ping He, Grace Lin, Jun Hong Xia, Fei Sun, Loong Chueng Lo, Felicia Feng, Ze Yuan Zhu, Gen Hua Yue

**Affiliations:** 1 Molecular Population Genetics Group, Temasek Life Sciences Laboratory, 1 Research Link, National University of Singapore, 117604 Singapore

## Abstract

**Background:**

High density linkage maps are essential for comparative analysis of synteny, fine mapping of quantitative trait loci (QTL), searching for candidate genes and facilitating genome sequence assembly. However, in most foodfish species, marker density is still low. We previously reported a first generation linkage map with 240 DNA markers and its application to preliminarily map QTL for growth traits in Asian seabass (*Lates calcarifer*). Here, we report a high-resolution linkage map with 790 microsatellites and SNPs, comparative analysis of synteny, fine-mapping of QTL and the identification of potential candidate genes for growth traits.

**Results:**

A second generation linkage map of Asian seabass was developed with 790 microsatellite and SNP markers. The map spanned a genetic length of 2411.5 cM, with an average intermarker distance of 3.4 cM or 1.1 Mb. This high density map allowed for comparison of the map with *Tetraodon nigroviridis *genome, which revealed 16 synteny regions between the two species. Moreover, by employing this map we refined QTL to regions of 1.4 and 0.2 cM (or 400 and 50 kb) in linkage groups 2 and 3 in a population containing 380 progeny; potential candidate genes for growth traits in QTL regions were further identified using comparative genome analysis, whose effects on growth traits were investigated. Interestingly, a QTL cluster at *Lca371 *underlying growth traits of Asian seabass showed similarity to the cathepsin D gene of human, which is related to cancer and Alzheimer's disease.

**Conclusions:**

We constructed a high resolution linkage map, carried out comparative mapping, refined the positions of QTL, identified candidate genes for growth traits and analyzed their effects on growth. Our study developed a framework that will be indispensable for further identification of genes and analysis of molecular variation within the refined QTL to enhance understanding of the molecular basis of growth and speed up genetic improvement of growth performance, and it also provides critical resource for future genome sequence assembly and comparative genomics studies on the evolution of fish genomes.

## Background

Most economically important traits are quantitative in nature and are determined by many genes and gene complex where are described as quantitative trait loci (QTL) [1]. Traditional methods of genetic improvement of quantitative traits have relied mainly on phenotype and pedigree information [1], which are easily influenced by environmental factors. Genetic markers have made it possible to detect QTL that are significantly associated with traits [2], and made selection more effective. Genetic response can be improved by including the QTL in marker-assisted selection, which is a method of selection that makes use of phenotypic, genotypic and pedigree data [3].

Linkage maps are essential for mapping QTL [4]. In the past, genotyping of many markers was expensive, therefore, specific experimental designs were developed to reduce the impact of having fewer markers on statistical power [1]. More recently, however, high throughput methods have been developed to genotype markers such as microsatellites [5] and single nucleotide polymorphisms (SNP) [6], which have significantly reduced the cost. Linkage maps have been constructed for a number of foodfish species, such as salmon [7], rainbow trout [8,9], catfish [10], tilapia [11], grass carp [12], common carp [13], Asian seabass [14], European seabass [15] and Japanese flounder [16], gilthead seabream *Sparus aurata *[17-19] using RAPD, AFLP and microsatellites. Only in a few species, linkage maps solely based on codominant DNA markers (microsatellites and SNPs) were constructed. Most linkage maps in food fish species are not dense in comparison to these linkage maps in model fish species (e.g. Zebrafish [20]), chicken [21], live stock species (e.g. cattle [22,23], and pig [24]) and agronomic plant species (e.g. barley, soybean, grapevine [25-27]). QTL mapping in foodfish species is still in its infancy [6]. Only in a few species, such as Asian seabass [6], salmon [28], tilapia [29], Japanese flounder [30], rainbow trout [31] and European seabass [32], QTL for growth, meat quality, stress and disease resistance have been mapped in large genomic regions due to lack of a high resolution linkage map.

Linkage map with sequence-based markers is also a platform for comparative genome studies [33-36]. Recent comparative genome analyses based on genetic maps have already provided new insights into genome organization, evolution, and function across different organisms [12,33,35,37]. For example, comparison of the *Caenorhabditis briggsae *genetic map and the *Caenorhabditis elegans *genome reveals extensive conservation of chromosome organization and synteny despite a very long divergence time (80 to 110 million years), suggesting that natural selection operates at the level of chromosomal organization [37]. In another study, a genetic linkage map of the blind Mexican cavefish *Astyanax mexicanus *has been successfully applied to predict candidate quantitative trait genes relating to rib number and eye size by anchoring cavefish QTLs to the zebrafish genome [36]. BLAST searches of sequences of mapped markers of grass carp against the whole genome sequence of zebrafish revealed substantial macrosynteny relationship and extensive colinearity of markers between grass carp and zebrafish [12]. Identification of conserved synteny blocks across fish genomes would help to unravel ancestral genome architecture of fish and transfer genome information from model fish species to non-model foodfish species.

Asian seabass, *Lates calcarifer*, also called Barramundi, is one of the important foodfish species. This species has been cultured for more than 20 years in brackish-water ponds and in recent years in floating cages. The global annual production of Asian seabass was currently 400,000 metric tons according to FAO statistics [38]. In the past few years, we started a breeding program for Asian seabass [39] and developed a number of genomic tools such as microsatellites [40-42], SNPs in genes [43], microRNA [44], a linkage map with 240 microsatellites [14], BAC and cDNA libraries [43,45,46] and a BAC-based physical map [44] to facilitate the selective breeding program. The linkage map has been used to map QTL for growth traits, and significant QTL for growth traits were mapped on linkage groups 2 and 3 [39,47]. However, due to the lack of markers in the QTL regions, it is impossible to map the QTL in smaller chromosomal regions. For fine mapping QTL, comparative analysis of synteny and searching for candidate genes in QTL region, a high-density linkage map is essential.

In this report we present a second generation linkage map of Asian seabass. The current updated version of the Asian seabass consensus linkage map is a considerable improvement compared with the previous version [14]. This linkage map allowed for carrying out comparative mapping of synteny between Asian seabass and *Tetraodon nigroviridis*, and enabled fine mapping of QTL for growth traits. In addition, we identified potential candidate genes in QTL for growth traits, and defined the phenotypic consequences of alternative candidate gene alleles.

## Results

### Identification and genotyping of DNA markers

A total of 4300 clones collected from libraries enriched for CA-, GA-, CAA- GACA- and GATA-microsatellites were sequenced in both directions. Two thousand and eight hundred clones contained microsatellites, yielding 1520 unique sequences. Among the 1520 sequences, 1280 had enough flanking regions for primer design. The first set of 920 primer pairs was used to amplify three parents from two reference families [14] for linkage mapping. Six hundreds and sixty primers were selected to genotype the two reference families including 96 individuals (3 parents and 93 offspring) due to the fact that these primers could amplify easily scorable PCR products. Among the 660 microsatellite markers, 280 were tetranucleotide microsatellites, which could be more easily scored than the di-nucleotide microsatellites (see Additional file 1). Ten SNPs in nine genes were genotyped by direct sequencing each individual in the two reference families.

### Linkage mapping

Of 851 informative markers including 240 markers mapped in the first generation linkage map [14], 822 markers were assigned to linkage groups by two-point linkage analysis with LOD scores >3.0 using CRIMAP (Green et al. 1990). From this, a total of 790 (97%) markers were mapped by multipoint linkage analysis. Among these markers, 53 initiated with LcaB were microsatellites isolated from BAC clones, 53 LcaE from ESTs and 10 SNPs from genes (Additional file 1). Details about primer sequences, GenBank accession number, annealing temperature for PCR, PCR product size, and locations of the 790 markers are summarized in Additional file 1.

**Figure 1:**
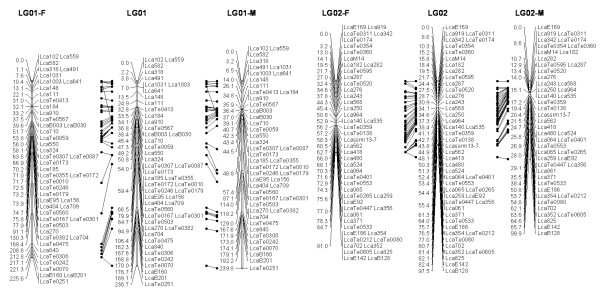
**The second generation linkage map of Asian seabass (LGs 1-2)**. Female (F) and male (M) maps are shown on the left and right, respectively, and the consensus map is shown in the center. The same loci are connected with solid lines.

**Figure 2:**
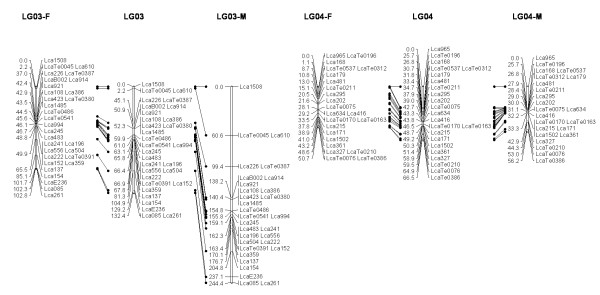
**The second generation linkage map of Asian seabass (LGs 3-4)**.

**Figure 3:**
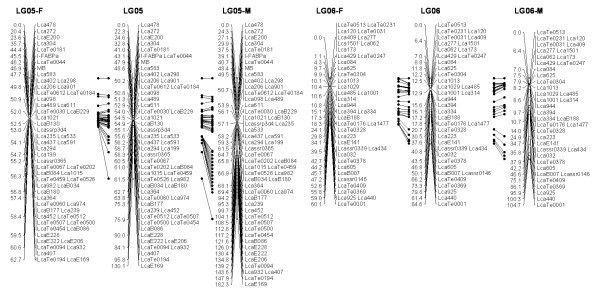
**The second generation linkage map of Asian seabass (LGs 5-6)**.

**Figure 4:**
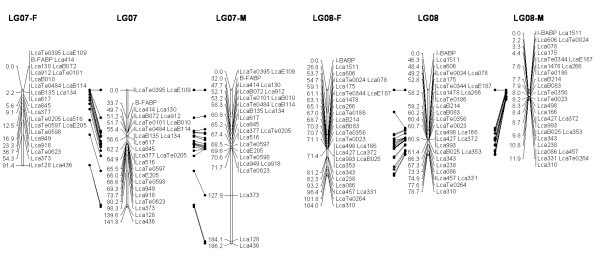
**The second generation linkage map of Asian seabass (LGs 7-8)**.

**Figure 5:**
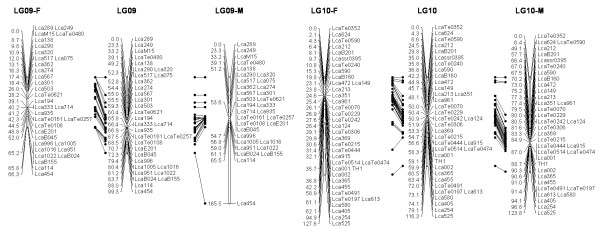
**The second generation linkage map of Asian seabass (LGs 9-10)**.

**Figure 6:**
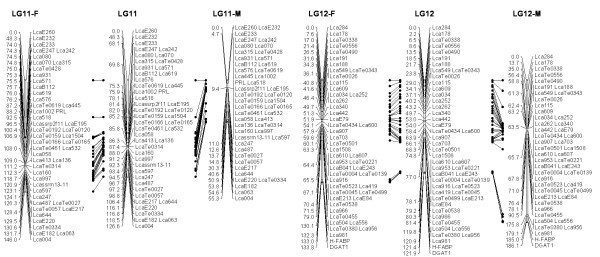
**The second generation linkage map of Asian seabass (LGs 11-12)**.

**Figure 7:**
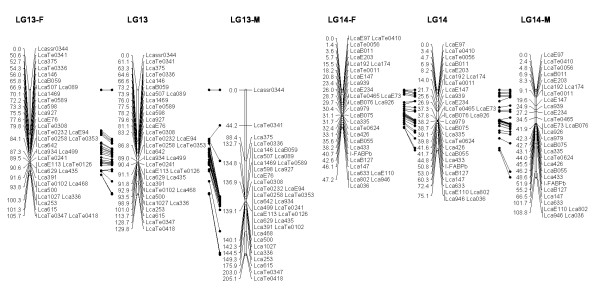
**The second generation linkage map of Asian seabass (LGs 13-14)**.

**Figure 8:**
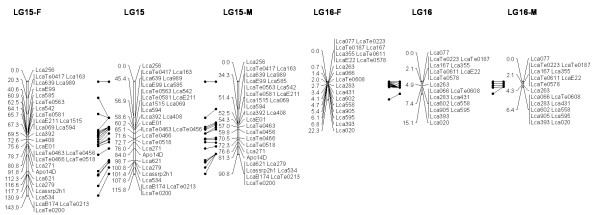
**The second generation linkage map of Asian seabass (LGs 15-16)**.

**Figure 9:**
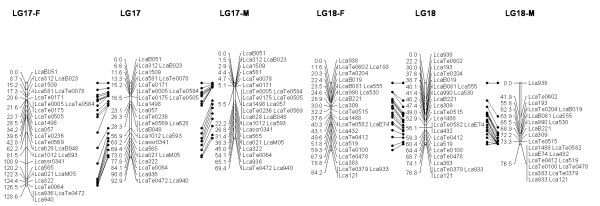
**The second generation linkage map of Asian seabass (LGs 17-18)**.

**Figure 10:**
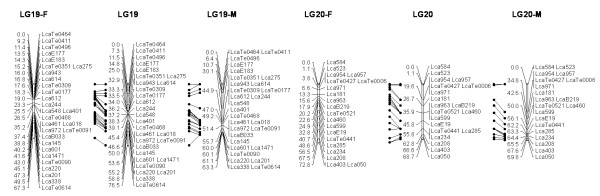
**The second generation linkage map of Asian seabass (LGs 19-20)**.

**Figure 11:**
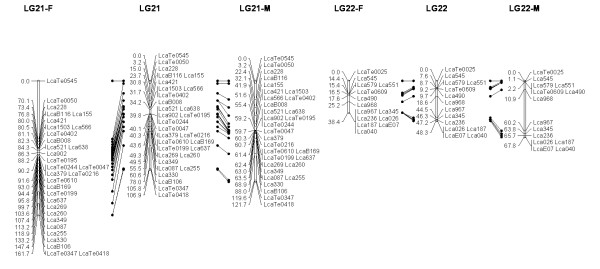
**The second generation linkage map of Asian seabass (LGs 21-21)**.

**Figure 12:**
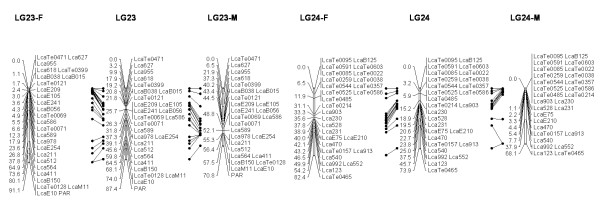
**The second generation linkage map of Asian seabass (LGs 23-24)**.

In most regions, the order of the markers on the new map was consistent with the previous map [14], although some regions were rearranged through incorporating the new markers and correcting old marker data. The current sex-averaged map spanned 2411.5 cM of the Asian seabass genome (Figure 1, 2, 3, 4, 5, 6, 7, 8, 9, 10, 11, 12). In the map, the intermarker distance was 3.0 cM. These 790 markers were located in 501 unique locations on the linkage map of the Asian seabass, with an average inter-location space of 4.8 cM (Table 1).

**Table 1:** Summary of the second generation linkage group of Asian seabass

LG	No. of Markers	Sex averaged		Female		Male	
		cM	cM/marker	cM	cM/marker	cM	cM/marker
1	48	236.7	4.9	225.6	4.7	239.8	5.0
2	49	97.5	2.0	91.0	1.9	99.9	2.0
3	31	132.4	4.3	102.8	3.3	244.4	7.9
4	23	66.5	2.9	50.7	2.2	56.2	2.4
5	58	130.1	2.2	62.7	1.1	182.3	3.1
6	40	84.6	2.1	60.1	1.5	104.7	2.6
7	27	141.8	5.3	91.4	3.4	186.2	6.9
8	29	78.7	2.7	104.0	3.6	11.9	0.4
9	33	99.3	3.0	66.3	2.0	165.5	5.0
10	37	116.3	3.1	127.6	3.4	123.8	3.3
11	48	126.6	2.6	146.0	3.0	55.3	1.2
12	51	133.8	2.6	121.9	2.4	186.1	3.6
13	36	129.8	3.6	105.7	2.9	205.1	5.7
14	30	75.1	2.5	47.2	1.6	108.8	3.6
15	30	115.8	3.7	143.0	4.6	90.8	2.9
16	19	15.1	0.8	22.3	1.2	6.4	0.3
17	28	92.9	3.3	128.6	4.6	69.4	2.5
18	24	76.8	3.2	84.2	3.5	76.5	3.2
19	29	76.5	2.5	67.3	2.2	63.3	2.0
20	20	68.7	3.4	72.8	3.6	69.8	3.5
21	31	106.9	3.4	161.7	5.2	121.7	3.9
22	14	48.3	3.5	38.4	2.7	67.8	4.8
23	27	87.4	3.2	91.1	3.4	70.8	2.6
24	28	73.9	2.6	82.4	2.9	68.1	2.4
Total	790/501*	2411.5	3.0/4.8**	2294.8	2.9	2674.6	3.4

LG: Linkage group; *: data is shown as number of markers mapped/unique locations; **: data is shown as cM/marker and cM/unique marker location

Sex-specific maps were also constructed. The length of the male map was 2674.6 cM, with an average intermarker distance of 3.4 cM whereas the female map was 2294.8 cM long, with an average intermarker spacing of 2.9 cM. The male map comprised linkage groups ranging in length from 6.4 to 244.4 cM while the female map contained linkage groups with a length ranging from 22.3 to 225.6 cM.

The distances between *LcaB201 *and *LcaTe0215 *at the end region of LG1, are 0 and 47.6 cM on female and male map respectively, while the distances between *Lca270 *and *LcaTe0382 *in the near middle region of LG1, possible proximal to centromere region, are 39.2 and 0 cM on female and male map respectively. Similarly, in the end regions between *LcaE142 *and *LcaB128 *on LG2, *Lca1508 *and *LcaTe0045 *on LG3, *LcaTe0194 *and *LcaE169 *on LG5 and *Lca114 *and *Lca454 *on LG9, females had much lower recombination rates in telomeric regions than males. While in the near middle regions, possibly within regions proximal to the centromere between *Lca359 *and *Lca137 *on LG3, *LcaB045 *and *Lca996 *on LG9, *Lca365 *and *LcaTe0191 *on LG10, *Lca1012 *and *Lca565 *on LG17, *Lca512 *and *Lca564 *on LG23, *LcaTe0485 *and *LcaTe0214 *on LG24, recombination rates were much higher in females. It showed that females have much lower recombination rates in telomeric regions than males, while recombination rates were much higher in females within regions proximal to the centromere.

### Comparative genome analysis

**Figure 13:**
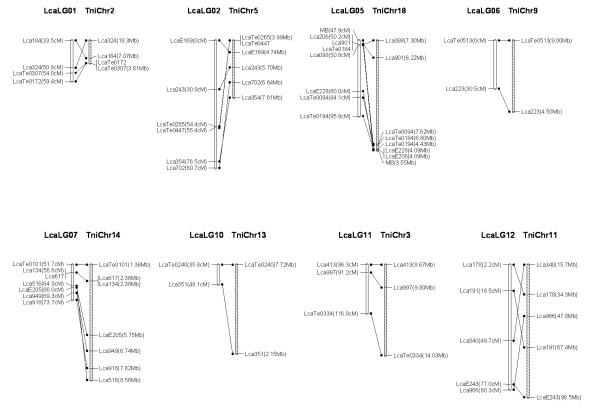
***Lates calcarifer *(Lca) linkage map and *Tetraodon nigroviridis *(Tni) synteny**. Homologous Lca (empty) and Tni (dashed) chromosomes are shown with lines connecting homologous markers.

**Figure 14:**
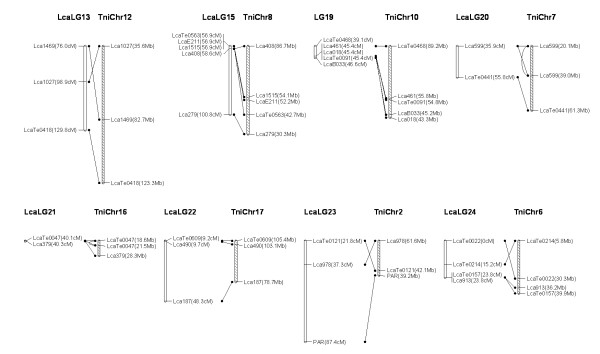
***Lates calcarifer *(Lca) linkage map and *Tetraodon nigroviridis *(Tni) synteny (continued)**. Homologous Lca (empty) and Tni (dashed) chromosomes are shown with lines connecting homologous markers.

We used BLAT to identify homologs of the sequence-based markers of *Lates calcarifer *on the map. Sixty seven markers had a homolog and could be assigned to the chromosomes of *Tetraodon nigroviridis*. We compared the *Lates calcarifer *genetic map with assembled genome sequences of *Tetraodon nigroviridis*, identifying conserved synteny blocks in 16 of the 24 *Lates calcarifer *linkage groups, each of which contained 3 to 8 markers. The largest synteny block conserved between *Lates calcarifer *and *Tetraodon nigroviridis *was found in LG5 with 8 markers spanning 48 cM in the *Lates calcarifer *linkage group and their best matches spanning 4 Mb in chromosome 18 of the *Tetraodon nigroviridis *genome (Figure 13, 14, Additional file 2). Blasting sequences of DNA markers of Asian seabass against whole genome sequences of zebrafish and medaka detected only very few conserved synteny (data not shown).

### Fine mapping QTL

**Figure 15:**
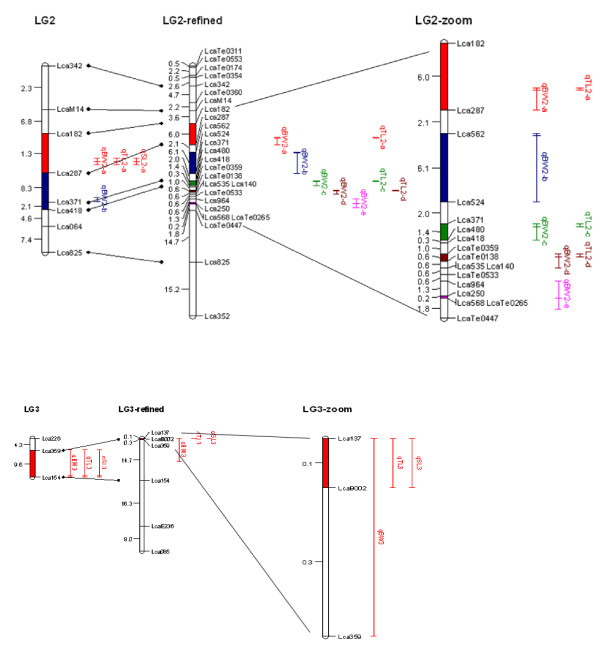
**QTL for growth traits identified on Asian seabass**. See Table 2 for details about the effects of QTL. The position of the QTL is indicated on the right of the linkage groups. The QTL bars indicate experiment-wise LOD support confidence interval in which the inner line indicates position of maximum LOD score. The highlighted region on LGs shows QTL interval between two flanking markers. The peaks of qBW2-a and BW2-b were flanked by *Lca182 *and *Lca287*, and closely linked to *Lca287 *with a distance of 2 cM. The peaks of the other QTL (qBW2-b, c, d, e and qBW3) were detected near the positions of markers *Lca562*, *Lca371*, *Te0359*, *Lca250 *and *Lca137*.

Additional 33 novel microsatellites located near QTL for growth traits on LGs 2 and 3 were selected and mapped with QTL panel containing 380 offspring. The linkage map was significantly improved for further QTL mapping as compared to the previous map by enhancing the density of markers for refinement of QTL positions (Figure 15). QTL analysis was carried out with genotype data of the markers and phenotypic data of all the progeny in the QTL mapping panel.

QTL affecting body weight, total length and standard length were identified on an experiment-wise scale. The experiment-wise LOD significance thresholds were 3.6, 5.5 and 5.4 for body weight, total length and standard length, respectively, while the linkage-group-wise LOD significance thresholds varied from 2.3 to 5.5 (Table 2). Eleven QTL controlling body weight, total length and standard length were detected on LGs 2 and 3. Multiple QTL Model (MQM) mapping with initial QTL did not change the results.

**Table 2:** Location of QTL and magnitude of QTL effects on growth traits in Asian seabass

Trait	QTL	LG	Position (cM)	CI (cM)	Flanking markers	Interval of flanking markers (cM)	Nearest marker and gene	Distance to the nearest marker (cM)	LOD	LOD threshold		PVE (%)	Phenotype means			
										Experiment-wise	Linkage group-wise		m1f1	m1f2	m2f1	m2f2
Body weight	*qBW2-a*	LG2	20.3	20.3-22.3	Lca182-Lca287	6.0	Lca287	2	4.9**	3.6	3.5	30.2	26.8	23.6	39.5	26.9
	*qBW2-b*	LG2	26.4	24.4-30.5	Lca562-Lca524	6.1	Lca562	0	4.0**	3.6	3.5	5.3	29.3	25.8	32.9	28.1
	*qBW2-c*	LG2	32.5	32.5-33.9	Lca371-Lca480	1.4	Lca371 in cathepsin D	0	4.5**	3.6	3.5	7.0	25.4	29.0	28.9	34.0
	*qBW2-d*	LG2	35.2	35.2-36.4	LcaTe0359-LcaTe0138	0.6	LcaTe0359	0	4.5**	3.6	3.5	5.3	33.0	28.4	29.1	25.7
	*qBW2-e*	LG2	39.1	37.6-40.1	Lca250-Lca568	0.2	Lca568 in KCTD15; LcaTe0265 in csmd2	0	4.0**	3.6	3.5	4.7	28.6	32.8	25.9	29.0
	*qBW3*	LG3	0	0-0.1	Lca137-LcaB002	0.1	Lca137	0	5.3**	3.6	2.6	6.3	31.8	25.1	32.3	28.0

Total length	*qTL2-a*	LG2	22.3	20.3-20.5	Lca182-Lca287	6.0	Lca287	2	5.5**	5.5	5.5	53.1	120.1	105.0	138.0	130.5
	*qTL2-c*	LG2	32.5	32.5-32.7	Lca371-Lca480	1.4	Lca371 in catheps D	0	5.5**	5.5	5.5	8.6	116.5	123.3	123.0	131.0
	*qTL2-d*	LG2	35.2	35.2-35.4	LcaTe0359-LcaTe0138	0.6	LcaTe0359	0	5.5**	5.5	5.5	6.5	129.6	121.8	123.2	117.5
	*qTL3*	LG3	0	0-0.1	Lca137-LcaB002	0.1	Lca137	0	5.7**	5.5	2.3	6.7	127.2	116.9	128.2	121.6

Standard length	*qSL2-a*	LG2	22.3	20.3-20.5	Lca182-Lca287	6.0	Lca287	2	5.1	5.4	5.3	53.8	98.7	86.7	114.2	108.1
	*qSL2-c*	LG2	32.5	32.5-32.7	Lca371-Lca480	1.4	Lca371 in catheps D	0	5.0	5.4	5.3	7.9	96.6	101.8	101.5	108.1
	*qSL2-d*	LG2	35.2	35.2-35.4	LcaTe0359-LcaTe0138	0.6	LcaTe0359	0	5.3*	5.4	5.3	6.2	107.1	100.6	101.8	97.3
	*qSL3*	LG3	0	0-0.1	Lca137-LcaB002	0.1	Lca137	0	5.7**	5.4	2.5	6.7	105.2	96.5	106.0	100.4

For each QTL detected, the confidence interval (CI), linkage group maximum LOD score, and percentage of the phenotypic variance explained (PVE) are indicated. The experiment-wise LOD significance thresholds are 4.5 for body weight, and 6.1 for total length and body length. Mean phenotypic values of each trait were also calculated for those progeny with the alternate alleles at the most closely linked microsatellite markers, inherited from the male parent (m1 or m2), alleles from the female parent (f1 or f2). **: Experiment-wise significant QTL; * Linkage-group-wise significant QTL.

The three QTL qBW2-a, qTL2-a and qSL2-a near the marker *Lca287 *showed high percentage of phenotypic variance explained (PVE) of 30.2, 53.1 and 53.8%, respectively (Table 2). These QTL together with qBW2-b are consistent to the previous QTL mapping results and their positions were finely refined as discussed later on.

Due to the more markers integrated in the QTL region, five QTL, i.e. qBW2-c, qBW2-d, qBW2-e, qTL2-c and qTL2-d, were newly detected. Although LOD values of qSL2-a and qSL2-c were not higher than the significant threshold, we still listed qSL2-a and qSL2-c to Table 2 as they showed obvious peaks and the LOD scores were very close to the threshold.

On LG2, the peaks of qBW2-a and qBW2-b were flanked by *Lca182 *and *Lca287 *with distance 6.0 cM, and closely linked to *Lca287 *with a distance of 2 cM. Similarly, the peaks of the other QTL (qBW2-b, c, d and e) were located in a small region between flanking markers, with intervals of 6.1, 1.4, 0.6, 0.2 and 0.1 cM respectively. On LG3, qBW3 was located in an interval of 0.1 cM between *Lca137 *and *Lca159*. The LOD peaks were detected near the positions of markers *Lca562*, *Lca371*, *LcaTe0359*, *Lca250 *and *Lca137 *respectively (Table 2, Figure 15).

### Potential candidate genes in refined QTL

**Figure 16:**
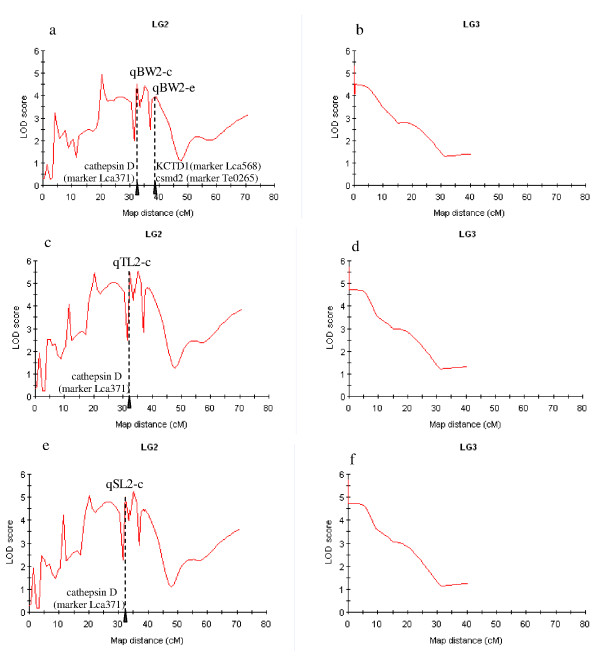
**Mapping of QTL for growth traits**. a, QTL for body weight on LG2; b, QTL for body weight on LG3; c, QTL for total length on LG2; d, QTL for total length on LG3; e, QTL for standard length on LG2; f, QTL for standard length on LG3. The lines were drawn by plotting the LOD scores at each marker as well as at 1.0 cM intervals along the linkage group. The arrow shows the position of genes and their markers where overlapped with QTL peaks.

As positions of some microsatellites collocating with QTL peaks, sequences of these DNA markers were blasted against the whole genome sequences of zebrafish and medaka. We detected some potential candidate genes for growth. Among these markers, a QTL cluster including qBW2-c, qTL2-c and qSL2-c were located in between markers *Lca371 *and *Lca480 *with distance of 1.4 cM, and QTL peaks collocated with the position of *Lca371*, whose sequence hit to genome sequences at 16.325 Mb on the chromosome 18 (Dr-18) in zebrafish, and at 2.311 Mb chromosome 3 (Ol-3) in medaka (Figure 16). When looking closely, we found that the sequence of the marker *Lca371 *showed very high similarity of the cathepsin D gene of medaka and zebrafish. We also found that the *Lca371 *showed similarity to the cathepsin D gene (Genbank accession nos: TC168775 and TC156330) of rainbow trout. Comparing the sequences of *Lca371 *to all 24,000 EST sequences generated by our group (unpublished data); we found that *Lca371 *was located in the cathepsin D gene of Asian seabass.

The peak of qBW2-e was located between the markers *Lca568/LcaTe0265 *and *Lca250 *with distance of 0.2 cM (Figure 16). The sequence of the marker *Lca568 *hit to genome sequences at 29.898 Mb on the chromosome 3 (Ol-3) in medaka, and some of the sequences of the marker *Lca568 *showed very high similarity of the *KCTD15 *gene of medaka. The sequences of *LcaTe0265 *hit to genome sequences at 35.626 Mb on the chromosome 19 (Dr-19) in zebrafish, and some of the sequences of the marker *LcaTe0265 *showed very high similarity of the *csmd2 *gene of zebrafish.

For *Lca562*, *LcaTe0359 *and *Lca137*, there were no hits to genes in medaka and zebrafish genomes.

### Effects of gene allele substitution

**Figure 17:**
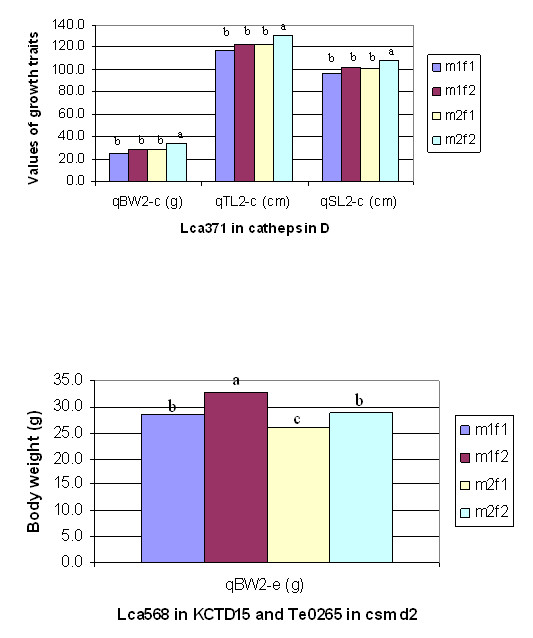
**The effects of genotypes of cathepsin D (left), *KCTD15 *and *csmd2 *(right)**. The letters on the top of bars indicate the level of difference in phenotypic value between genotypes. The same letter indicates that the difference is statistically insignificant, whereas different letters represent statistically significant (Bonferroni T tests at α *<*0.01) difference.

A two-way ANOVA was performed on the 380 progeny using four allelic combinations (m1f1, m1f2, m2f1 and m2f2) from markers in the three candidate genes of cathepsin D, KCTD15 and csmd2 in order to investigate associations between traits and genotypes of these genes. The phenotype values of each allelic combination of these three genes are listed in Table 2 and Figure 17. Significant differences of phenotype means among different allelic combinations were identified, revealing the effects of alternate gene alleles inherited from the parents. Progeny with m2f2 genotype at the marker *Lca371 *located in cathepsin D, showed the highest phenotype values. Similarly, progeny with m1f2 genotype at the marker *Lca568 *located in *KCTD15 *and *LcaTe0265 *in *csmd2*, showed the highest phenotype values. These results suggested the effect of these genes on growth related traits.

## Discussion

### Map density and recombination rate

The linkage map presented in this paper represents one of the most dense linkage maps based on microsatellites and SNPs for foodfish species. This second generation linkage map of Asian seabass contained 3.3 times as many sequence-based co-dominant loci as did the previous linkage map [14], and more sequence-based co-dominant markers than the linkage maps of major foodfish species, such as salmon [7], tilapia [11], common carp [13], grass carp [12], Japanese flounder [16], catfish [10] and European seabass [15]. However, in comparison to the linkage maps of model fish species (e.g. zebrafish [20]), livestock (e.g. cattle [22,23], pig [24]), chicken [21]) and agronomical plant species (e.g. rice, barley, soybean, grapevine, [25-27,48], the marker density of the Asian seabass linkage map is still lower. This is mainly because the whole genome sequence and large number of SNPs in Asian seabass are still not available. Fortunately, sequencing the whole genome of Asian seabass is in agenda. The third generation of linkage map could be based on large number of SNPs. The current Asian seabass map spanned 2411.5 cM, and it is estimated to span much more of the Asian seabass genome in comparison with the previous linkage groups [14]. It had a resolution of 3.4 cM, which is sufficient for fine mapping QTL for future marker-assisted selection.

The average recombination rate across all linkage groups is approximately 3.4 cM/Mb in Asian seabass, which is higher than that in zebrafish (1.35 cM/Mb) [20], catfish (1.65 cM/Mb) [10], tilapia (1.3 cM/Mb) [11], grass carp (1.2 cM/Mb) [12], human (1.20 cM/Mb), mouse (0.5 cM/Mb) [49] and lower than the plant Arabidopsis thaliana (5 cM/Mb; calculated based on data from The Arabidopsis Information Resource website). Based on the genome size of 700 Mb [14,50] we estimated the average intermarker distance to be approximately 0.88 Mb in the new linkage map which consisting 790 markers. This suggests that QTL, if identified, can be narrowed down to rather small genomic regions. The enhanced map will be invaluable not only for QTL and gene mapping but also for comparative genome analysis and assembling the Asian seabass genome once the whole genome sequencing available.

### Sex-specific patterns of recombination

Different recombination rates between the sexes have been reported in mammals [8] and other fish species [15,17,51] with female map distances usually greater than those in male maps [8,52]. In Japanese flounder, the recombination rate was unusually higher in males (7.4 times) compared to females [16]. Different to the previous map [14], the overall sizes of the male and female maps are comparable (female: 2294.0 cM; male: 2674.6 cM). Females had much lower recombination rates in telomeric regions than males, while recombination rates were much higher in females within regions proximal to the centromere. Females have much lower recombination rates in telomeric regions than males on LGs 1, 2, 3, 5 and 9, while recombination rates are much higher in females within regions possibly proximal to the centromere on LGs 1, 3, 9, 10, 17, 23 and 24. This is similar to linkage map of rainbow trout [8].

### QTL fine mapping

To improve the utility of the QTL in MAS, and to move toward the positional cloning of candidate genes, fine mapping of the QTL to a smaller region of the chromosome is necessary [53,54]. In foodfish species, although QTL mapping has been conducted in a few species, such rainbow trout [31,55], salmon [56], Japanese flounder [30] and tilapia [29], QTL were mapped in large genomic regions, usually bigger than 10 cM. In Asian seabass, the number of available markers on our previous map [14] limited the possibility for fine mapping QTL in Asian seabass; therefore the development of additional markers in the QTL regions is an important target in this study. As compared to the previous QTL mapping results, the positions of QTL were much refined with the markers from the enhanced map (Figure 15). The peaks of qBW2-a and BW2-b were flanked by *Lca182 *and *Lca287 *with distance reduced from 11.3 cM on previous map to 6.0 cM, and closely linked to *Lca287 *with a distance of 2.0 cM. Similarly, the peaks of the other QTL (qBW2-b, c, d and e) was refined from 8.3 cM on previous map to a small region between flanking markers, with intervals 6.1, 1.4, 0.6, 0.2 and 0.1 cM respectively. On LG3, qBW3 was from 9.6 cM to 0.1 cM. The refined QTL supply a basis for identifying potential candidate genes located in these refined QTL through comparative genome analysis. Fortunately, the complete genome sequences of several mode fish species (i.e. zebrafish, medaka, freshwater pufferfish, fugu and stickleback) [57-60] are available, and the sequencing of genomes of several important foodfish species (common carp, salmon and tilapia) are in progress.

### Comparative genome analysis

Methods to map sequence based markers and to compare maps with relevant model systems are crucial to extend genomic-level analysis to non-model species [54]. According to the phylogenetic tree including over 30 fish species, constructed using 12 mitochondrial genes in our previous study, Asian seabass is more closely related to *T. nigroviridis *than to zebrafish and medaka [61]. However, in our previous comparative mapping, no synteny block could be identified between Aisan seabass and *T. nigroviridis *although flanking sequences of 55 microsatellites showed high similarity to known genomic DNA sequences of *T. nigroviridis *[14]. Herein, we developed our second generation linkage map with high density and demonstrated that, 16 syteny blocks of Asian seabass were syntenic to the 16 counterparts of *T. nigroviridis *chromosomes. The result showed certain colinearity for the 16 syntenic chromosome/linkage pairs between the two foodfish species, *T. nigroviridis *and Asian seabass. The conserved syntenies identified here between the Asian seabass and *T. nigroviridis *should facilitate studies on genome evolution and analysis of structural genome, but more importantly should facilitate functional inference of genes in Asian seabass. It is well known that determination of gene functions is difficult in non-model species; functional genome analysis will have to rely heavily on the establishment of homologies from model species. Mapping more ESTs or gene sequences on the linkage map of Asian seabass should enhance comparative mapping, thereby transferring genome information from model species to Asian seabass.

### Identification of candidate genes in QTL

Maps with sequence-based markers are useful not only for comparative genomics but also resolving mapped genomic regions to a tractable number of candidate genes, especially if there is synteny with related model species. After refining the positions of QTL, the regions of candidate genes were delimited more precisely. The LOD peaks were detected near the positions of markers *Lca562, Lca371, LcaTe0359, Lca250 *and *Lca137 *respectively, therefore we examined regions of the sequenced zebrafish and medaka genome corresponding to the QTL regions on Asian seabass, using the markers overlapped on QTL LOD peaks. Such an approach allows us to prioritize our research effort on candidate genes that are found collocating with QTL LOD peaks. Candidate gene approaches have been successfully used for identification of QTL [62,63]. Three putative growth-related genes, cathepsin D, *KCTD15 *and *csmd2*, were found to be positioned directly at QTL peaks between small region of 0.2-1.4 cM, making them the strongest candidate genes for growth traits. Further two-way ANOVA revealed that allelic substitution at these two genes showed significant effects on growth-related traits. Certain allelic combinations showed significantly higher values of the growth traits. The candidate genes are rare examples of QTL fine mapping in foodfish species. Interestingly, the QTL cluster at *Lca371 *underlying growth traits of Asian seabass showed similarity to the cathepsin D gene of human, which is related to cancer [64] and Alzheimer's disease [65] and has become a hotspot of human genetics study. The genotypes at these genes may be useful for growth improvement through marker-assisted selection, gene cloning and functional analysis. In humans, *KCTD15 *may be associated with obesity [66], while *csmd2 *may be an oligodendroglioma suppressor [67]. Therefore it is interesting to further study the mechanisms underlying the associations between polymorphisms in these two genes with growth traits in Asian seabass.

## Conclusions

We constructed a second generation genetic linkage map and carried out comparative mapping of synteny between Asian seabass and *T. nigroviridis*. Moreover, we applied this map for refinement of QTL for growth traits using extensive progeny testing with defined recombination within the QTL region. We estimated their locations within short intervals, identified potential candidate genes, and further defined the phenotypic consequences of alternative candidate gene alleles. This second generation linkage map should facilitate the advancement of genetic studies for a wide variety of complex traits in foodfish species. In the future, more SNP markers should be identified by using next generation sequencing [68] to enable whole genome association studies [69] to facilitate genomic selection [70] and to understand the genetic basis of phenotypic variation of important traits [71,72].

## Methods

### Reference families for linkage mapping and QTL analysis

The reference families used for the construction of the first generation linkage map [14] were used for the construction of the second generation linkage map. Briefly, a whole broodstock containing 94 brooders, including 48 males and 46 females collected from the wild in Southeast Asia four years ago, were genotyped with nine polymorphic microsatellites as described previously [14,41]. One female and two male brooders were selected for constructing a mapping panel because of their high allelic diversity and genetic differences. By crossing the female and two male brooders, millions of eggs were produced. A total of 47 and 46 full-sib progeny were randomly collected from the two full-sib families, respectively. The reference family including one parental pair and 380 offspring used for preliminary QTL analysis for growth traits was used in the fine mapping of QTL for growth traits.

Fin clips of the parents were collected and kept in absolute ethanol, whereas the whole body of each offspring at the age of 90 days post hatch (dph) was cut into small pieces, soaked in absolute ethanol, and kept in a -80° freezer. DNA was isolated and arrayed into 96-well PCR plates as described preciously [14].

### Identification and genotyping of DNA markers

Partial genomic DNA libraries enriched for CA-, GA-, CAA-, GACA- and GATA- repeats were constructed as described previously [73]. Repeat-enriched DNA fragments of 400-1200 bp in length were cloned into pGEM-T vector (Promega, San Luis Obispo, CA), and transformed into XL-10 blue supercompetent cells (Stratagene, La Jolla, CA). The libraries were arrayed into 96- well plates for bidirectional sequencing on an ABI3730xl DNA sequencer (ABI, Foster City, CA) using the BigDye V3.0 kit and M13 and M13 reverse primers. Redundant and overlapping sequences were grouped using Sequencher (Gene Codes, Ann Arbor, MI). Unique sequences were compared to known microsatellite sequences of Asian seabass prior to primer design to remove redundancy. Microsatellites in ESTs were isolated using a method described previously [74]. Sequences containing CA>7, GA>7, CAA>6, GACA>5 and GATA>5 were subjected to primer design using PrimerSelect (DNASTAR, Brighton, MA), targeting a product size between 100 and 400 bp. Primers were designed for each unique sequence using PrimerSelect (DNASTAR, Madison, WI). One primer of each pair was labeled with FAM or HEX fluorescent dyes at the 5'-end. PCR for genotyping was carried out as described previously [14]. Products were analyzed using the DNA sequencer ABI3730xl, and genotyping was carried out to determine fragment size against the size standard GS-ROX-500 (Applied Biosystems, Foster City, CA) with software GeneMapper V3.5 (Applied Biosystems).

SNPs in genes were detected by PCR amplification of DNA of three parents and sequencing of PCR products. Briefly, genes of Asian seabass were aligned with genomic sequence data from zebrafish on GenBank. Primer sites in conserved exon regions were identified and primer pairs allowing PCR amplification of an intron-spanning fragment were developed. Amplified intron-spanning fragments which were sequenced as described above to detect SNP in the three parents from the reference families for linkage mapping. Ten SNPs were detected in 9 genes (Additional file 1). The genotyping for the 10 polymorphic SNP markers were performed by direct sequencing PCR products using ABI 3730xl Genetic Analyzer (Applied Biosystems). SNP genotypes were scored using Sequencher 4.9 (Genecodes).

### Linkage Analysis

CRIMAP 2.4 software [75] was used for linkage analysis. Markers were placed into linkage groups based on twopoint LOD scores (>3.0) with markers from the previous map. Ordering the markers within a linkage group began with the previous map. A new marker was inserted into the map by evaluating the LOD at every possible location. The marker was then inserted at the location with the best LOD, and the change in length of the linkage group was evaluated. If a switched pair improved the LOD, the pair was switched and the process repeated until no better LOD could be found. The process of switching the order of marker pairs was repeated to finalize marker order. The final maps represent the most likely marker order identified with the complete data set. Once the most likely order had been derived, sex-average and sex-specific linkage distances were estimated for each using the Kosambi function. MapChart 2.2 software was used for graphical visualization of the linkage groups [76]. The total length of the linkage map was calculated by summing up the length of all 24 linkage groups.

### Comparative genome analysis

As our previous study showed that Asian seabass is more closely related to *T. nigroviridis *than to zebrafish and medaka [61], comparison of flanking sequences of each Asian seabass marker on the map with the assembled genomic DNA sequences of the *T. nigroviridis *was conducted using BLAT http://www.genoscope.cns.fr/blat-server/cgi-bin/tetraodon/webBlat. BLAT searching was performed with a score above 80 and an alignment length of more than 50 bp as recommended [77].

### QTL fine mapping

In our previous studies [39,47], we identified significant QTL for growth traits on LGs (linkage groups) 2 and 3. To map the QTL on LGs 2 and 3 with more precision, 33 additional microsatellites located near the QTL on these two linkage groups were genotyped. With genotype data of the markers on the QTL regions and phenotypic data of the 380 progeny, QTL analysis was carried out using the program MapQTL 5.0 [78]. Interval mapping and multiple QTL model (MQM) mapping were utilized to detect any significant association between growth-related traits and marker loci in the data sets. Cofactors for MQM analyses were automatically selected with a p-value of 0.02. The LOD score significance thresholds were calculated by permutation tests in MapQTL 5.0, with a experiment-wise significance level of α < 0.05, n = 1000 for significant linkages. Calculation of the percentage of phenotypic variance explained (PVE) by a QTL was performed in MapQTL 5 on the basis of the population variance found within the progeny of the cross.

### Potential candidate genes and their effects on growth traits

For those microsatellites, positions of which were detected well-overlapping with QTL peaks, we did a blast search of the sequences of microsatellites to identify potential candidate genes in these loci. Blast searches were done against the whole genome sequences of zebrafish and medaka in the current ENSEMBL release version http://www.ensembl.org and against all known sequences in GenBank.

A two-way ANOVA was performed on the 380 progeny using four allelic combinations (m1f1, m1f2, m2f1 and m2f2) from markers in the three candidate genes in order to investigate associations between phenotypic traits and genotypes of these genes. Mean phenotypic values of each trait were calculated for those progeny with the alternate alleles of the microsatellite markers, inherited from the male parent (m1 or m2), alleles inherited from the female parent (f1 or f2). This was conducted by using the general linear model (GLM) procedure of SAS (SAS Institute) and the Bonferroni method of multiple comparisons with α < 0.01.

## Abbreviations

QTL: Quantitative trait loci; SNP: Single nucleotide polymorphism; cM: Centimorgan; RAPD: Rapid amplified polymorphic DNA; AFLP: Amplified fragment length polymorphism.

## Authors' contributions

GHY initiated and overviewed the Asian seabass project and finalized the manuscript. WCM and YGH designed the study. WCM, BZY, HXP, XJH, SF, LLC, FF and ZZY conducted the experiments. WCM conducted the data analysis and wrote the manuscript. All authors have read and approved the final manuscript.

## Supplementary Material

Additional file 1 **790 DNA markers mapped to the second generation linkage map of Asian seabass**. Click here for file

Additional file 2 **Comparative mapping of markers of *Lates calcarifer *through BLAT search against *Tetraodon nigroviridis *genome**. Click here for file
