# Supplementary material for: A high-resolution linkage map for comparative genome analysis and QTL fine mapping in Asian seabass, Lates calcarifer
Source: BMC Genomics. 2011 Apr 2;12:174. doi: 10.1186/1471-2164-12-174 (PMC3088568; doi:10.1186/1471-2164-12-174)
Supplement: Additional file 2 — Comparative mapping of markers of Lates calcarifer through BLAT search against Tetraodon nigroviridis genome. [file 1471-2164-12-174-S2.DOC]

**Additional Table 2 Comparative mapping of markers of *Lates calcarifer* through BLAT search against *Tetraodon nigroviridis* genome**

| **Number** | ***Lates calcarifer* Linkage group** | **Name** | ***Tetraodon nigroviridis* chromosome** | **Identity**  **(%)** | **Alignement length** | **score** |
| --- | --- | --- | --- | --- | --- | --- |
| 1 | LG01 | LcaTe0307 | chr2 | 97.10 | 103 | 97 |
| 2 | LG01 | LcaTe0172 | chr2 | 99.00 | 100 | 98 |
| 3 | LG01 | Lca184 | chr2 | 89.10 | 427 | 177 |
| 4 | LG01 | Lca324 | chr2 | 95.90 | 101 | 89 |
| 5 | LG02 | LcaTe0265 | chr5 | 85.30 | 240 | 167 |
| 6 | LG02 | LcaTe0447 | chr5 | 81.30 | 211 | 137 |
| 7 | LG02 | LcaE169 | chr5 | 86.60 | 264 | 130 |
| 8 | LG02 | Lca243 | chr5 | 84.50 | 344 | 181 |
| 9 | LG02 | Lca702 | chr5 | 89.60 | 115 | 91 |
| 10 | LG02 | Lca354 | chr5 | 89.50 | 115 | 90 |
| 11 | LG05 | MB | chr18 | 85.60 | 521 | 297 |
| 12 | LG05 | LcaE206 | chr18 | 88.00 | 227 | 88 |
| 13 | LG05 | LcaE228 | chr18 | 87.00 | 115 | 85 |
| 14 | LG05 | LcaTe0194 | chr18 | 87.00 | 115 | 85 |
| 15 | LG05 | LcaTe0184 | chr18 | 76.30 | 406 | 196 |
| 16 | LG05 | LcaTe0094 | chr18 | 91.60 | 417 | 87 |
| 17 | LG05 | Lca901 | chr18 | 91.40 | 207 | 171 |
| 18 | LG05 | Lca098 | chr18 | 100.00 | 91 | 86 |
| 19 | LG06 | Lca223 | chr9 | 84.00 | 210 | 161 |
| 20 | LG06 | LcaTe0513 | chr9 | 88.40 | 203 | 93 |
| 21 | LG07 | LcaTe0101 | chr14 | 82.20 | 190 | 123 |
| 22 | LG07 | Lca617 | chr14 | 76.50 | 129 | 82 |
| 23 | LG07 | Lca134 | chr14 | 87.50 | 184 | 108 |
| 24 | LG07 | LcaE205 | chr14 | 97.00 | 215 | 188 |
| 25 | LG07 | Lca949 | chr14 | 85.60 | 244 | 168 |
| 26 | LG07 | Lca918 | chr14 | 87.50 | 586 | 161 |
| 27 | LG07 | Lca516 | chr14 | 78.70 | 228 | 106 |
| 28 | LG10 | Lca351 | chr13 | 89.60 | 105 | 83 |
| 29 | LG10 | LcaTe0240 | chr13 | 91.70 | 269 | 218 |
| 30 | LG11 | Lca413 | chr3 | 84.30 | 173 | 135 |
| 31 | LG11 | Lca997 | chr3 | 92.80 | 96 | 82 |
| 32 | LG11 | LcaTe0334 | chr3 | 90.90 | 174 | 142 |
| 33 | LG12 | Lca340 | chr11 | 87.80 | 220 | 116 |
| 34 | LG12 | Lca178 | chr11 | 80.60 | 359 | 116 |
| 35 | LG12 | Lca966 | chr11 | 83.90 | 234 | 142 |
| 36 | LG12 | Lca191 | chr11 | 89.20 | 321 | 123 |
| 37 | LG12 | LcaE243 | chr11 | 91.70 | 186 | 149 |
| 38 | LG13 | Lca1027 | chr12 | 89.60 | 144 | 114 |
| 39 | LG13 | Lca1469 | chr12 | 89.10 | 347 | 193 |
| 40 | LG13 | LcaTe0418 | chr12 | 90.80 | 184 | 157 |
| 41 | LG15 | Lca279 | chr8 | 85.10 | 146 | 90 |
| 42 | LG15 | LcaTe0563 | chr8 | 78.30 | 168 | 80 |
| 43 | LG15 | LcaE211 | chr8 | 88.70 | 116 | 105 |
| 44 | LG15 | Lca1515 | chr8 | 88.90 | 728 | 196 |
| 45 | LG15 | Lca408 | chr8 | 77.60 | 159 | 90 |
| 46 | LG19 | Lca018 | chr10 | 77.70 | 136 | 99 |
| 47 | LG19 | LcaB033 | chr10 | 89.20 | 319 | 123 |
| 48 | LG19 | LcaTe0091 | chr10 | 89.00 | 772 | 178 |
| 49 | LG19 | Lca972 | chr10 | 87.30 | 286 | 130 |
| 50 | LG19 | Lca461 | chr10 | 77.90 | 209 | 112 |
| 51 | LG19 | LcaTe0468 | chr10 | 88.20 | 313 | 98 |
| 52 | LG20 | Lca599 | chr7 | 83.30 | 175 | 134 |
| 53 | LG20 | Lca599 | chr7 | 96.80 | 100 | 85 |
| 54 | LG20 | LcaTe0441 | chr7 | 89.60 | 351 | 232 |
| 55 | LG21 | LcaTe0047 | chr16 | 81.00 | 274 | 86 |
| 56 | LG21 | LcaTe0047 | chr16 | 89.40 | 222 | 103 |
| 57 | LG21 | Lca379 | chr16 | 78.70 | 239 | 96 |
| 58 | LG22 | Lca187 | chr17 | 90.00 | 181 | 141 |
| 59 | LG22 | Lca490 | chr17 | 90.10 | 547 | 114 |
| 60 | LG22 | LcaTe0609 | chr17 | 87.10 | 217 | 161 |
| 61 | LG23 | PAR | chr2 | 85.00 | 220 | 97 |
| 62 | LG23 | LcaTe0121 | chr2 | 88.90 | 1323 | 392 |
| 63 | LG23 | Lca978 | chr2 | 89.00 | 394 | 276 |
| 64 | LG24 | LcaTe0214 | chr6 | 84.50 | 504 | 200 |
| 65 | LG24 | LcaTe0022 | chr6 | 97.80 | 88 | 84 |
| 66 | LG24 | Lca913 | chr6 | 86.30 | 136 | 105 |
| 67 | LG24 | LcaTe0157 | chr6 | 84.60 | 369 | 206 |
